# Memetic/Metaphorical Digital Twins: Extending Knowledge Co-Creation Across Economics, Architecture, and Beyond

**DOI:** 10.3390/biomimetics11030220

**Published:** 2026-03-18

**Authors:** Ulrich Schmitt

**Affiliations:** 1 Stellenbosch Business School, P.O. Box 610, Bellville 7535, South Africa; schmitt@knowcations.org; 2 Knowcations, Start-Up at La Plage Factory, 32 St. Georges Street, Port Louis 11324, Mauritius

**Keywords:** Memetic/Metaphorical Digital Twins (MDT), Cognitive Digital Twins (CDT), Digital Community Platform, Knowledge Management (KM), Personal Knowledge Management System (PKMS), Knowledge Co-Creation, Boundary Objects (BO), Memetics, biomimetics, entropy, economics, architecture, design science research (DSR)

## Abstract

This article introduces Memetic/Metaphorical Digital Twins (MDTs) as a novel extension of Digital Twin typologies by twinning conceptual schemes, complementing Industrial, Human, and Cognitive Digital Twins. MDTs embed cultural, organizational, and semiotic knowledge into digital frameworks, enabling the recombination and evolution of knowledge structures across disciplines. Drawing on Schlaile’s economic perspectives and Mavromatidis’s architectural lens of entropy and constructal thermodynamics, this study demonstrates how MDTs can address systemic challenges in communication, knowledge transfer, and design. A Digital Community Platform, under development for supporting decentralized Personal Knowledge Management Systems (PKMS), provides the operational foundation, integrating iterative KM cycles to support knowledge co-creation. Its logic and logistics substitute the traditional document paradigm with a memetic approach by utilizing memes as replicable, adaptive knowledge units, thereby mimicking biological evolution and ecosystem resilience in digital platform environments. It aims to offer distributed, decentralized, bottom-up, affordable, knowledge-worker-centric applications prioritizing personalization, mobility, generativity, and entropy reduction; its mission is to serve a knowledge-co-creating community characterized by highly diverse individual Abilities, Contexts, Means, and Ends (ACME) facing increasingly volatile, uncertain, complex, and ambiguous futures (VUCA). A Boundary Object Taxonomy to Omnify Memetic Storytelling (BOTTOMS) is proposed to further structure atomic units of meaning—such as memes, mythemes, narratemes, and reputemes—into a unified framework for authorship and dissemination. The article situates MDTs within a design science research paradigm, outlines current implementation progress, and identifies future developments, including AI-supported curation, personalized metrics, and expanded boundary objects. Together, these contributions position MDTs as a universal framework for adaptive, transdisciplinary knowledge co-creation.

## 1. Digital Divides and Wicked Problems: Toward Memetic/Metaphorical Digital Twins

Despite the rapid diffusion of digital technologies, their promised dividends remain unevenly distributed worldwide [1]. The Inner Development Goals (IDG), aligned with the UN Sustainable Development Goals (SDG), emphasize interventions to reduce widening opportunity divides and strengthen connections among knowledge and people [2]. However, current web-based systems often impose rigid entry, exit, and transfer barriers, draining users’ attention, time, productivity, and resources. For SMEs (small and medium-sized enterprises) and individuals, organizational knowledge management systems remain financially inaccessible.

This general plea for focusing on tackling wicked challenges and spaces (as exemplified above for life-long learning interventions to improve problem-solving capabilities by applying the IDGs in SDG contexts) has also been expressed, for example, in the areas of economics, architecture, and knowledge management (KM). Wicked problems are ill-defined, contradictory, and dynamic, with complex interdependencies. Crucially, the information required to understand them depends on the proposed solution itself [3]—a dynamic that resonates strongly with biomimetic approaches.

This article unfolds in three parts. Part 1 situates the proposed KM concept within recent calls for path creation in economics and architecture. Part 2 elaborates the Memetic/Metaphorical Digital Twin (MDT) within a digital KM platform under development and as a distinct addition to Digital Twin categories, since MDTs link to virtual rather than physical counterparts. Part 3 synthesizes perspectives across domains to demonstrate how MDTs can advance biomimetic approaches to knowledge co-creation.

Part 2 follows up on a 2025 paper of the International Conference on Cooperative Design, Visualization and Engineering (CDVE) that proposed expanding Digital Twin categories to include MDTs [4]. While key arguments (and content of 8%) are retained, this article further details the integration of MDTs and Cognitive Digital Twins (CDTs) within a digital platform, supported by expanded visual mappings and illustrative application scenarios. This approach mirrors how ecosystems process information and evolve collaboratively.

This work complements an ongoing longitudinal design science research (DSR) project and related academic publications (Parts 1 and 3 and, partially, Part 2), which have guided the evolution of artifacts and design decisions/theories in support of complexity awareness, co-creation skills, knowledge, and social connectedness. With this article, the MDT’s potential is not just focused on the PKM application but amplified by exemplifying its applicability in other domains.

By combining conceptual analysis, synthesis, design science research, and systems thinking, this study adopts a transdisciplinary methodology tailored to wicked problems. Conceptual analysis and synthesis provide theoretical grounding and integration of perspectives across economics, architecture, and knowledge management. Design science research guides the iterative development of the digital platform, while systems thinking ensures coherence across complex interdependencies. This methodological framing not only strengthens the rationale for MDTs but also reflects biomimetics’ emphasis on adaptive, cross-domain problem-solving and the transfer of biological strategies into organizational and technological contexts, as elaborated in Part 3.

The aim of this article is to demonstrate the utility of MDTs within the digital platform under development, emphasizing their capacity to bridge economics, architecture, and KM through biomimetic principles. This common need for structured, reusable knowledge frameworks and content serving trans-disciplinary communities connects closely with biomimetics’ research interests in the related fields of practices [5], ontologies [6], standards [7], education [8], and ethics [9].

The article focusses first on the areas of evolutionary economics (Section 2) and architecture (3), introduces some essential DT notions (4), before visualizing and detailing DT-related aspects of the personal KM approach (5) and the cooperative design/engineering perspective (6), to make the case for a new category hosting metaphorical or memetic DTs (7). It further focuses on applicability and utility to demonstrate the relevance of the MDT innovation by repurposing and synthesizing concepts from semiotics for the memetic space (8) before concluding in Section 9.

## 2. Memetics in Evolutionary Economics and Systemic Change

Schlaile et al. adopt memetic notions to reintroduce memes into the study of economics [10]. By adapting the memeticists’ and Popperian three-world notions (to be referred to in Section 5.1), they are focusing on the meme’s eye view (to complement the agent-centered view) and its social connectivity dynamics fostering creativity, emerging innovations, as well as evolving organizational cultures (framed by the five interdependent ‘i’s of economemetics: information, instruction, imitation, innovation, and interconnection [10] (p. 9).

In his book “Memetics and evolutionary economics: To boldly go where no meme has gone before” [11], Schlaile draws on Popper’s World:123 framework. Based on his earlier work [12], he distinguishes memes as types:

• E (environmental/artifactual representations in the physical world, W:1);
• I (individual mental accounts in W:2, shaping instructional cultures and influencing material ‘m’-cultures [13]);
• P (primal, genuine objects or abstract ‘bits’ in W:3, only replicable once shared.

Aligned to Beinhocker [14], Schlaile identifies p-type memes as constraining a culture’s opportunity space [11] (p. 41), thereby shaping the evolution of enabling rules, routines, and tools. Expanding digital spheres are providing new memetic niches for the ‘e-i-p’-types and ‘i-m’-cultures, yet truth, trustworthiness, and utility—including equitable distribution of digital dividends [1]—remain uncertain.

As “entities of complex population systems” and potential “building blocks of an organizational culture”, memeplexes may evolve into complex networks, with nodes representing memes and edges depicting compatibility relations [11] (pp. 45, 75). In the platform’s knowledge graph, these nodes host content, container, context, and craft memes to be associatively edge-indexed via connector memes (across all p/e/i/m sections).

Although Schlaile does not mention entropy explicitly, he highlights the attention-consuming nature of thriving memes and warns of risks including degradation, fragmentation, siloism, bias, and undesired path dependencies or lock-ins.

For organizational benefit, he cautions, that the memetic interconnective and interdependent potentials can only be realized at an appropriate level of cognitive distance, meaning “that agents can only learn from each other and innovatively utilize the knowledge they exchange if their cognitions are neither too similar nor too different” [11] (p. 105); Section 6.5 introduces a career-spanning six-persona-level-heuristic to address these transitional concerns. If compatible, their shared mental memetic representations become “quasi-agents” that afford “actions and behaviors”—a term (affordances) aligned with Gibson’s ecological theory of affordances [15,16]—such as creating boundary objects (BO) (filters, heuristics, or interpretative schemata) “for discovering, creating, and exploiting opportunities” [11] (pp. 105, 106).

As a bridge between disciplinary divides and/or as a transitional state between ill-structured and not yet well-structured representations (e.g., standards or infrastructure), BOs afford diverse social actors shared collaborative spaces of common understanding. BOs aim for more generic approaches (1) fitting wider classes of tasks and problem spaces, (2) accommodating interpretative flexibility across diverse peers, and (3) enabling clients to repurpose and re-contextualize knowledge for personal and local circumstances [17,18].

Having also acknowledged the memetic significance for system entrepreneurship and innovation systems [19] as well as absorptive capacity (defined as an individual’s or firm’s “ability to recognize the value of new information, assimilate it, and apply it to commercial ends” [20]) and systemic change processes, Schlaile proposes systematically exploring conceptual complementarities and synergies between remix practices (copy, transform, combine) “and established approaches to knowledge creation in evolutionary economics and organizational science as an avenue for future research” [11] (pp. 85, 189). In the platform, atomic memes change as any three-way-combination: content (its text), container (its animate or inanimate host), or contexts (its tags or topic associations) with its versions and paths stored in as-built genealogies.

Schlaile’s memetic framing of evolutionary economics—emphasizing systemic change, remix practices, and the dynamics of memeplexes—provides a conceptual foundation for extending Digital Twin categories. It supports the rationale for MDTs as knowledge structures that can capture, recombine, and evolve cultural and organizational patterns in ways analogous to biological and ecosystem processes

## 3. Entropy, Ontology, and Urban Spaces: An Architectural Lens

While Schlaile’s memetic framing in evolutionary economics highlights systemic change and the dynamics of cultural opportunity spaces, similar challenges and opportunities emerge in architecture and urban spaces, which likewise grapple with wicked problems, complex interdependencies, and the need for adaptive knowledge structures. Mavromatidis is grounding his analysis in physics and entropy as defined by the Gouy–Stodola theorem. Through this lens, entropy—defined as a thermodynamic measure of irreversibility and energy dissipation in open systems—manifests as disorder and inefficiency in complex systems—such as digital architectural contexts, where it may hinder synthesis under constructal law and exergy principles [21] of spaces, materials, and functions into creative, resilient, and adaptive designs [22] (to manifest as positive entropy or generativity [23,24]).

In proposing a paradigm shift towards enabling ‘living’ architecture settings, he presents buildings and cities as dynamic, self-regulating, organic open systems interconnected by efficient energy and resource flows, governed by constructal thermodynamics, which—for guiding efficient outcomes—require deliberate systemic interventions, supported by smart digital toolkits that enable analysis, projectability, and world-making. While projectability “provides a forward-looking means to frame the future” by adjusting “both the technology and the context” through design knowledge [25] (p. 3), worldmaking offers a metaphor for people’s approaches to narrowing the gap between their local actual world and desired possible worlds through building capacities, artifacts, and bonding experiences [26].

As key actors, Mavromatidis portrays architects “as creators, scientists, readers and interpreters of forms”, guided by visions that transcend physical and conceptual limitations and reimagine urban existence [22] (p. 2). From the outset, creators’ artistic spaces of potential generativity exist as positive entropy, contrasting with the rigid order of Taylor’s scientific management principles [27]) able to envisage and design “manmade systems [and artifacts] akin to biosystems to regulate themselves, maintain their own functions, and interact with their environment in a self-directed manner” [22] (p. 4).

Subsequent processes need to assimilate a diversity of constraints, including, for example, environmental affordances, material qualities, stakeholders’ interests, prior knowledge/experience, or verbalized records (e.g., manuals, standards, laws, practices, or established conceptual frameworks). Any consideration reflected on may limit the initial solution potential and risk confusion if not properly communicated, producing disorder as negative entropy through errors, delays, and unmitigated risks. Any agreed design decisions further restrain “subsequent stages in ways that cannot simply be undone without significant effort or loss of creative intent” [28] (p. 11) and may also lead to path-dependencies.

Mavromatidis equates these processes (like his interpretation of urban spaces) to thermodynamic flow systems with “transient states within a continuum of possibilities” which “adapt to optimize circulation, connectivity, and resource distribution” striving to “evolve over time to facilitate greater access to their flow”. Any action or decision, hence, represents thermodynamic “work performed on a system [shifting] its configurational or relational energy (and entropy)” and balancing order and disorder through dynamic equilibrium states in a “one-way [irreversible] progression of design evolution”. These scenarios are all driven by the laws of nature but the two architectural contexts addressed also by the “shared experiences and contributions of countless people, each adding their voice to the ongoing narrative, rather than being confined to a singular, prescriptive idea of what architecture ought to represent” and where “opposing forces and influences” converge as managed milestones of accepted “clarity and stability” towards cohesive visions and outcomes [28] (pp. 2, 6, 7, 11, 12).

Accordingly, his advice is to transcend relevant internal boundaries and restrictions by integrating outward-looking value-adding opportunities to attend to the “larger, interconnected whole (the future urbanite)” allowing for harmonizing efficiency with excess, tradition with innovation, and equilibrium with disruption, all while carving a path toward spaces that resonate with meaning and vitality” [28] (pp. 12, 14). In such a context, the as-built memetic genealogies referred to keep on capturing each shared meme’s usage across knowledge assets by applying Usher’s cumulative synthesis principles [29], a process-oriented performative account of innovation. Rather than meta-tagged fragmented documents, the higher memetic granularity facilitates traceability and repurposing of knowledge across time, distance, disciplines, and voices for more rapid iterative dissemination and improvement.

There is no mention of memetics at all, but Mavromatidis bases his entropic interpretations instead on the notion of mythemes [30]. Mythemes—alongside memes, narratemes, biographemes, reputemes, motifs, and topos—function as irreducible minimal units of analysis for decoding complex cultural systems into their smallest meaningful parts and identifying recurring patterns—analogous to boundary objects (BO) (Section 8).

In need of a meta-language able to address the semantic challenges of architectural narratives to base cohesive, intricate structures on discrete, elementary parts, he commends mythemes for their ability to escalate relational (subject-predicate) dynamics from granular micro (mythemes) to higher-order macro (mythos) states for facilitating energetic and informational exchanges. He commends Greimas’s Semiotic Square [31] as a deductive model, whose junctions, disjunctions, and transformations correspond to dynamic energy fluxes in architectural and urban systems. He further demonstrates the utility of Greimas’s actantial model (sender, receiver, helpers, opponents) [32] for structuring and rationalizing architectural narratives.

Mavromatidis’s emphasis on entropy, constructal thermodynamics, and mythemes as minimal cultural units provides a complementary lens to Schlaile’s memetics. Together, they substantiate the rationale for MDTs as transdisciplinary knowledge structures capable of capturing, recombining, and evolving both economic and architectural patterns in ways resonant with biomimetic principles.

## 4. From Industrial and Human to Cognitive Digital Twins: Foundations for Memetic/Metaphorical Extensions

By situating architecture and urban spaces within thermodynamic and semiotic frameworks, Mavromatidis provides a complementary lens to Schlaile’s memetics. Together, these perspectives underscore the need to revisit Digital Twin typologies. Section 4 introduces essential notions as a foundation for positioning Memetic/Metaphorical Digital Twins (MDTs) within the broader landscape of evolving twin categories, segmented into four archetypes and pathways (Table 1).

Biomimetics-related DTs have been explored in robotics and manufacturing (IDTs), medicine and motion studies (HDTs), and neuropsychology (CDTs). Cognitive Mimetics (CM) provides a methodological bridge by mapping human information processing onto intelligent technologies [36], with implications for both HDTs (human–machine collaboration) and CDTs (knowledge explication and expertise sharing) [37].

Despite these advances, realizing the vision of CDTs remains constrained by knowledge management challenges. Representation is hindered by fragmented or inaccessible tacit knowledge; acquisition is complicated by scattered documents; and updating is obstructed by neglected expansions, unlearning, and evolutionary processes [38].

Additional barriers emerge when DTs operate within shared digital platforms, including issues of data quality, ownership, interoperability, costs, opportunism, trust, and legal constraints. Empirical studies have identified recurring challenges for IDTs—dependency, uncertainty in data management, varying customer needs, insufficient work methods, and unsuitable payment models—alongside management strategies such as transparency, incentive models, control, and servitization [39].

While Industrial, Human, and Cognitive Digital Twins provide essential foundations, their limitations in representing and evolving cultural knowledge highlight the need for Memetic/Metaphorical Digital Twins (MDTs). Therefore, Section 5 visualizes and details DT-related aspects of personal knowledge management in some more technical detail, making the case for MDTs as not only desirable but also implementable distinct new category.

## 5. From PKMS to Knowledge Co-Creation: Toward CDTs and MDTs

**Figure 1:**
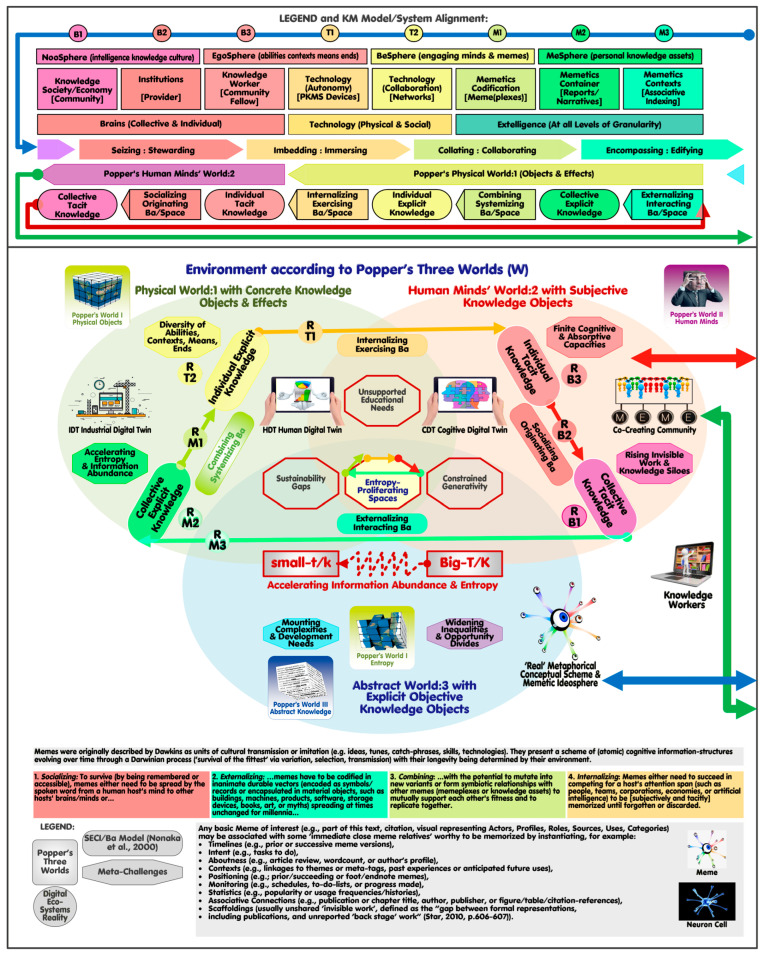
Ecosystems of digital platforms for knowledge co-creation with KM models and Digital Twins (CDTs and MDT) Memetic Ideosphere as left mirror image of Figure 2: Digital Twin, [41].

**Figure 2:**
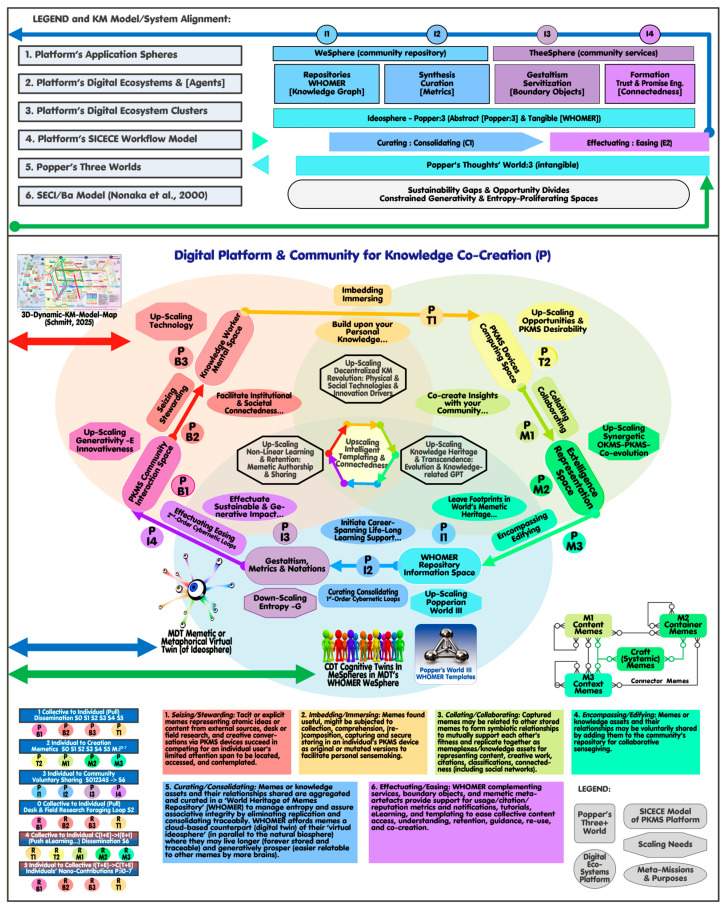
Ecosystems of digital platforms for knowledge co-creation with KM models and Digital Twins (CDTs and MDT); Digital Twin as right mirror image of Figure 1: Memetic Ideosphere, [4,41].

A digital platform for knowledge co-creation can be envisioned as autonomous community members, each supported by decentralized KM devices, linked to their centralized (and locally mirrored) CDT-‘MeSpheres’. Members may voluntarily share personal content with the community’s collaborative, centralized ‘WeSphere’ to be iteratively consolidated, curated, and made accessible in a continuous feedback loop by the provider [4]. Levy describes personal KM “as the elementary process that makes possible the emergence of the distributed processes of collective intelligence, which in turn feed it” [40] (p. 116). The colored rectangles at the bottom of Figure 1 and Figure 2 provide a more detailed account of the co-evolution of memes and their hosts outside (SECI-cycle Figure 1) and within (SICECE-cycle Figure 2) the MDT.

This notion aligns with cooperative engineering, an approach that develops products (documents and knowledge assets [representing “nonphysical claims to future value or benefits” [42] (p. 96)]) and supports production structures (like memetics) and processes (like authorship) across multi- and trans-disciplinary contexts with strong stakeholder interaction [43] (p. 360).

Memetics originated from Dawkins’ book ‘The Selfish Gene’ in which he introduced Memes as units of cultural transmission or imitation and as a driver of human evolution (by complementing genes). Memes present a metaphorical scheme of (atomic) cognitive information structures that evolve over time through a Darwinian process of variation, selection, and transmission [44]. From the meme’s-eye view, every human is a machine for making more memes, a vehicle for propagation, an opportunity for replication, and a resource to compete for. But memes exist only virtually and have no intentions of their own; they are merely information pieces in a feedback loop with their longevity being determined by their environment [45,46,47].

While this “new information technology landscape is giving us complete new challenges, [it also provides] new opportunities that we did not expect before” [48] (p. 305). With some of the challenges and barriers already exemplified, further conceptual symmetries and design strategies need to be taken care of.

The system analyses included, for example, a Strengths/Vulnerability/Intervention-Assessment related to Digital Threats (SVIDT) [49], Visioneering [50], and Benchmarking against the DIN ISO 30401:2018-KMS Standard [51,52].

Moreover, sixteen dynamic KM-related models (as empirically/conceptually validated human information processing ‘sources’) have been integrated, and all are synthesized and visualized as a three-dimensional ‘public-transport-like’ map; it provides the backdrop for a layover depicting seven user application scenarios (knowledge acquisition, creation, and sharing) [53].

### 5.1. General Visual Overview with Positioning of Key Elements (Figure 1 and Figure 2) 

Cognitive Mimetics (CM) mapping relations relevant to the DT perspective are visualized in Figure 1 and Figure 2, which depicts the system environment in the environment (Figure 1) and its virtual platform counterpart (Figure 2) as a mirror image. The common denominators are the three interlocked circles on each side [4] representing Popper’s three-world notion (referred to in Section 2 by Schlaile) [54]. Standing in for the sixteen dynamic KM models [53], Nonaka’s SECI (socialization, externalization, combination, internalization) and Ba (spaces) models (left) [41] serve as counterparts to the platform’s novel SICECE (seizing, embedding, collating, encompassing, curating, effectuating) model (right), aligning knowledge worker practices with digital workflows. The top-layer provides the legend and logic (horizonal iterative flows) how the SECI/Ba model (6), Popper’ three world notion (5), the platform’s SICECE workflow model (4) are aligned according to the Platform’s digital ecosystem clusters (3), digital ecosystems with exemplified agents (2), and spheres of application (1) to be detailed later.

The visual (Figure 1 and Figure 2) summarizes key aspects presented in prior publications, like the current meta-challenges (left hexagons) as well as anticipated platform scaling needs (right hexagons) and related meta-missions and purposes (right ellipses). IDTs and HDTs are depicted as icons in their relevant Popperian Worlds (left) with CDTs and MDT located in Popper’s World:3 (right), linked to their real-world counterparts (left) represented by the platform’s co-creating community and the metaphorical memetic ideosphere (left). The memetic relevance is described in line with the uniquely referenced (small circles) SECI and SICECE steps (bottom). A thumbnail map icon at the top (middle) points to the 3D ‘public-transport-like’ map [53] with their seven user application scenarios alluded to earlier (to be referenced as S0–S6).

The gray rectangle (Figure 1 bottom) further summarizes what a meme may represent depending on its platform-specific type (content, container, context, craft, or connector meme type), whose relations are depicted on the right of Figure 2.

### 5.2. Inner Workings with Detailed Visualized Workflows (Figure 3)

**Figure 3:**
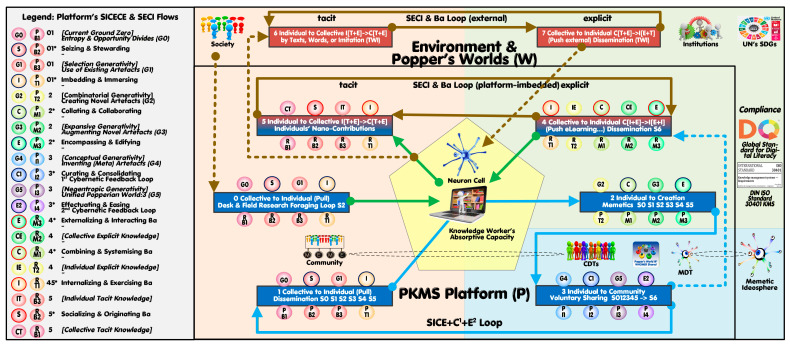
Key flows and segments for co-creating community members (based on WHOMER, MDT, and CDTs) (see a fully dissected 3D map showing memetic/cognitive path scenarios aligned to the topography of 16 dynamic KM models [51,53].

The platform’s first four (decentralized) iterative steps align reversely to the SECI cycle (Figure 3, right vs. left) while two added (centralized) flows represent the platform provider’s tasks and services. The novel SICECE cycle is empirically and conceptually backed by the ‘Notional Model of the Sensemaking Loop for Intelligence Analysis’ [23,55] as well as the ‘Extended Concept-Knowledge Design Theory’ (CKDT) [24,28].

The centralized CDTs (people, colored icon right) representing the community member’s individual ‘MeSpheres’ (people, colored icon left) are accessible by the decentralized PKMS devices and their external SECI (left cycle) and its resonating digital SICE (right cycle) iterative dynamic workflows.

The semantic SICE-SECI-similarity has been adopted as an ‘emergent innovation strategy’ [56] due to the high failure risk of KMS implementations and radical innovations. The naming symmetry provides not only familiarity aiding acceptance but also promotes the significant synergies between the conventional organizational and the novel personal KMS approaches as an enabler for anticipated fruitful co-evolutions [4] (to be alluded to later).

### 5.3. Sensemaking and Sensegiving via the World Heritage of Memes Repository (WHOMER)

The voluntarily shared sections of the member’s CDT-‘MeSpheres’ are cumulatively synthesized in the centralized community’s collaborative ‘WeSphere’ referred to as the World Heritage of Memes Repository (WHOMER) [4], located in Popper’s World:3 [blue icon right].

The PKMS replaces the conventional document-centric storage paradigm, which replicates content via copy-paste operations, with a memetic approach that embeds and reuses digital document components via structural references [57] (p. 391). The PKMS’s continuous iterative sensemaking and sensegiving SICE cycles are constructing chronological chains (as-built genealogies) of original memes and their revised versions to create memeplexes, knowledge assets, and corpora; its centralized CE (Curation and Effectuating) services—after eliminating redundancies and fusing traceabilities—aggregate the shared content and safeguard the associative memetic integrity [4]. In the process, Popper’s objective-abstract (but intangible and inaccessible) Thought-World:3 is progressively transformed into its digital concrete, tangible, and accessible counterpart (further detailed in Section 7).

Figure 3 extracts and reorganizes the workflows transitioning from the worlds-vs-platform perspective (Figure 2 bottom left) to an individual knowledge worker perspective (Figure 3), with his/her optional interventions displayed as eight aggregated value chains (with some referencing the further detailed S0–S6 scenarios depicted in the 3D-map mentioned [53]).

The letters of the acronyms SECI and SICECE represent their flows as shown in the legend of Figure 3 (marked with a preceding asterisk *’). Flows represent the continuous dynamic transformation processes with Ba (or spaces) describing their context-specific features. The circulating evolving substances in the iterative cycles accumulate as stocks in the form of knowledge types (tacit-explicit/collective-individual as in SECI [41]) or progressing generativity types (as briefly defined in the SICECE legend [24,28]) (shown all in italics within square [] brackets).

Figure 3 (top) depicts the (externally operating) SECI cycle with its individual-to-collective (6) and collective-to-individual (7) value chain segments; community members in their personal environmental contexts engage by sharing knowledge or via learning (brown short-dashed arrows). The SICECE cycle is shown in three segments (bottom) with knowledge being transferred from WHOMER’s ‘WeSphere’ to community members’ MeSpheres’ (1), being selected, studied, augmented, complemented, and potentially voluntarily shared (2), to be centrally curated in a first and second cybernetic feedback loop (3).

Segment 0 represents the foraging loop, where community members search and acquire (pull) information from external sources [55] (brown short-dashed arrows). This knowledge is captured (green arrow) in PKMS devices and managed as an abduction process (from void, cues/clues, draft, to final for memes to be developed/adapted by the user). Segment 4 exemplifies the platform’s application of SECI’s collective-to-individual (push) segment internally to provide, for example, metrics, notifications, and e-Learning affordances. Segment 5 adopts the SECI’s individual-to-collective segment to portray how community members may engage in co-creating with their acquainted peers via the platform space; it adds a new constructual perspective to be relevant later in the economics and architecture contexts.

By integrating PKMS, SECI, and SICECE cycles into a collaborative platform, Section 6 demonstrates how Cognitive Digital Twins (CDTs) can support personal and collective knowledge co-creation. It portrays Memetic/Metaphorical Digital Twins (MDTs) as a distinct new category, extending Digital Twin typologies into the cultural and semiotic domain.

## 6. The Cooperative Design/Engineering Perspective

This section situates cooperative design and engineering within a memetic perspective, showing how MDTs extend existing practices and address sustainability challenges.

### 6.1. Memetic Parallels to Supply Chain Management (SCM)

This ‘memetic’ type of genealogical connectivity portrayed is already applied at the core of modern manufacturing and supply chain management (SCM), where the materials, labor, processes, and logistical resources needed for producing any good are digitally described and combined as bills of labor or materials [4]. But memes are not reduced when consumed, nor diminished when transferred or shared. Once captured, they remain permanently indexed and accessible. This genealogical indexing of memes parallels the logic of MDTs, which extend cooperative design practices into cultural and semiotic domains. MDTs reduce entropy and support iterative co-creation across disciplines, complementing industrial and cognitive twin categories.

### 6.2. Entropy and Sustainability Challenges

Knowledge processing, like other resources, can improve sustainable supply chain visibility. Yet it is also affected by digital e-waste, where regulations aim to promote the reuse and recycling of information to reduce resource consumption and overload [58] (pp. 1518, 1524). This entropy is further amplified, for example, as Industrial Symbiosis methodologies are facing barriers due to the current shortfalls of conventional KM systems and practices, where the novel memetic approach may provide a remedy [59].

### 6.3. Platform Benefits for Cooperative Design

The platform benefits also serve the recent recommendations “towards the realization of resource-efficient cooperative engineering and development systems” [60] (p. 744). The same applies to the findings of studying SMEs’ requirements for resilient cyber-physical production systems, which prioritized managing internal knowledge (including training materials and best practices) and adopting ICT solutions that are easy to use, standardized, secure, and interoperable [61] (p. 1616).

In cooperative design [62] and as exemplified by the 3D map, the platform documentation makes extensive use of visual language in the form of metaphors and shared mental models (over 100 figures published).

Compared to the document-centric paradigm, associatively indexed memes provide a much higher granularity (forming a sequence within a conventional document referencing other external memes from published documents as well as unpublished ‘scaffolding’ memes voluntarily shared by the authors via the platform). They enable detailed referencing, reduce entropy, accelerate iterative improvements, and provide usage, citation, and reputation metrics [4].

### 6.4. Value Propositions

These features illustrate how MDTs extend cooperative design by embedding cultural and organizational knowledge into digital frameworks, ensuring both resilience and adaptability. The platform’s value propositions can be interpreted through a memetic lens, where memes serve as carriers of absorptive, generative, functional, psychological, economic, and transformative roles (Table 2); they illustrate how memetic indexing enhances cooperative design and knowledge co-creation by improving granularity, reducing entropy, and enabling detailed usage, citation, and reputation metrics.

### 6.5. Collaboration and Cooperation

Although the platform focuses on its communities’ diverse Abilities, Contexts, Means, and Ends (ACME), it does not aim for substituting conventional organizational KMS (OKMS) but—due to its complementing participatory bottom-up approach—for a fruitful co-evolution.

A review of empirical KMS literature has provided details that allow a constructive structuring of this ambition. The study focused on changes in specific outcomes between three organizational core elements and identified and clustered the causing knowledge transfers. The authors have provided a comprehensive inventory of 248 distinct independent variables (actions, elements, and contexts), 108 dependent variables, and 61 unique theoretical concepts linked to clusters of five knowledge outcomes and four behavioral actions [63]. The relevant entities identified are repurposed below (in brackets) to augment the platform’s SICECE cycle (already depicted in Figure 1 and Figure 2, top) (Table 3).

### 6.6. Co-Evolution Spaces

With its educational agenda under development, a platform also qualifies as a multi-stage, career-spanning development tool (with meme-based knowledge assets repurposable as learning assets aligned to IDG/DQ/SDG criteria). The ACME-based personas to be served (linked to the value propositions in Table 2) are segmented into two triple-progressions: the first triple focuses on the individual and his/her motivation to utilize community-shared content for fostering their own personal knowledge and ambitions (1. students and lifelong learners, 2. researchers, and 3. authors). While continuing to benefit from these affordances, the personas of the second triple stages (4. educators, 5. projecteers, and 6. change makers) step outside their personal comfort zones for effectuating entrepreneurial, institutional, transcendental, or altruistic impacts, including empowering others.

**Figure 4:**
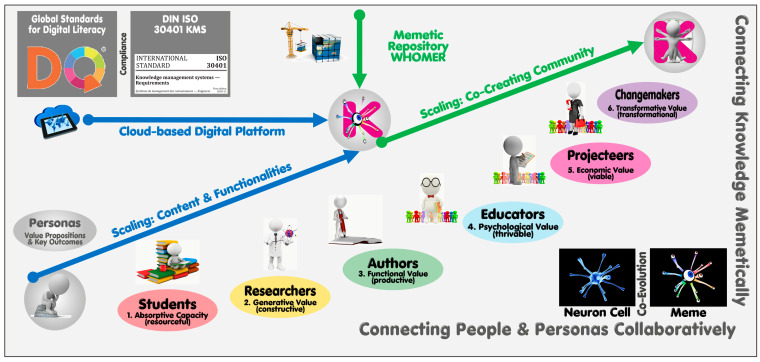
PKMS’s connectedness for scaling a co-creating community platform, [51].

As animate meme containers, the users generatively engage with their inanimate siblings (memetic representations of captured knowledge voids earmarked for further exploration, as well as physical/conceptual objects with their explicated/encapsulated knowledge) via the memes, treated as living organisms. The platform’s WHOMER knowledge base aims to mimic the memes’ metaphorical habitat (ideosphere) rather than prioritize the human biosphere. The memetic treatment of everything creates synergies benefitting standardizable structures, coding, processing, and user experiences. This focus, with its much higher knowledge granularity, fuses the intelligence of individuals and communities (CDTs) with the accumulating shared and stored extelligence of WHOMER’s meme heritage pool, twinning the ideosphere (MDT). From the scaling perspective, suitably incentivized and appreciative community members in pursuit of superior affordances come together to form a growing and vibrant co-creating community (Figure 4). An article is currently under review to break these affordances down in a persona-oriented 48-cell innovation appreciation matrix.

## 7. WHOMER as Memetic Ideosphere and MDT Enabler

This section positions WHOMER as an MDT enabler, showing how it functions as a knowledge heritage archive, transforms Popper’s World Three, and embodies the ideosphere metaphor.

### 7.1. WHOMER as Knowledge Heritage Archive

Supported by CDTs and centralized curation, WHOMER is envisaged as a single, digital (cloud-based), accumulating (community-shared novel and historic content), unified (transdisciplinary), negentropic (redundancy-eliminating) knowledge heritage archive [66]. As in the case of complex supply chains, statistics can be employed for advancing the metrics supporting the academic citation, innovation, and reputation economy, as well as servitization.

### 7.2. Transformation of Popper’s World Three

Simultaneously, Popper’s objective–abstract, intangible, inaccessible, and non-interrogatable philosophical–theoretical World Three is transformed into its concrete, tangible, accessible, and interrogatable WHOMER counterpart as its own ‘virtual twin’ twinned with the metaphorical scheme of the memetic ideosphere.

### 7.3. Memes and the Ideosphere Metaphor

Knowledge and its management are abstract concepts with no clearly delineated structure and no ‘real world’ referent. To apply structure and make them comprehensible requires the mapping of familiar notions of other disciplines to the one to be illuminated by means of analogical thinking and graspable metaphors [67].

The metaphor of memes implies that they resemble living organisms that survive by self-replicating via mental storage in human hosts, by influencing their hosts’ behavior to promote their further replication either by being communicated to others, explicated in records, or encapsulated in physical objects [4]. Thus, memes and their inbuilt ideas may be imagined as populating a memetic ideosphere as their habitat of operation (in parallel to the biosphere), where they are competing for attention [11] (pp. 47, 48) to survive.

### 7.4. WHOMER as MDT-Enabling Agent

Through PKMS’s logic and logistics, WHOMER operates within the same ideospheric space as its Digital Twin counterpart. Captured memes remain preserved and findable, and can evolve through learning and authoring processes via symbiotic relationships with other memes. PKMS’s WHOMER provides a space where memes can persist, adapt, and co-create (and boldly go where they may live long and prosper) by synergetically and simultaneously developing individuals’ capacity and repertoire for sharing, collaboration, and innovation.

This type of Memetic or Metaphorical Digital Twin (MDT) neither fits the IDT (not physical) nor the HDT (not human) nor the CDT (not real) categories introduced in Section 4 (Table 2). The MDT is twinning instead of what has been termed a conceptual scheme (seeking usefulness rather than truth) [68]. However, the WHOMER-MDT undeniably increases the potential of memes to mutate into new variants or memeplexes for mutually supporting each other’s fitness and flourishing together in a virtual MDT ideosphere [4].

WHOMER exemplifies the MDT category by twinning the conceptual scheme of the ideosphere, enabling memes to evolve into new variants and memeplexes that flourish together in a digital habitat. The following sections aim to strengthen these concepts and affordances by aligning them to Schlaile’s economic [11] and Mavromatidis’s architectural/semiotic [22] contexts and publications.

In Summary, the PKMS with its WHOMER exemplifies a new DT category: the Memetic/Metaphorical Digital Twin (MDT). Unlike conventional Digital Twins that replicate physical assets, biological systems, or organizational processes, this category uniquely twins conceptual schemes—memes, knowledge structures, and cultural narratives—into persistent, generative, and personalized digital entities. By enabling the twinning of meaning systems rather than material systems, the platform expands Digital Twin theory into the domain of cognition and culture. This new category is distinguished by:

• Conceptual Twinning: Replicating and evolving knowledge frameworks and cultural memes.
• Knowledge-Worker Centricity: Prioritizing personalization, generativity, and entropy reduction in knowledge flows.
• Distributed Architecture: Supporting decentralized, bottom-up co-creation of knowledge ecosystems.
• Dual Cognitive–Memetic Layering: Bridging individual reasoning structures (MeSphere) with collective cultural patterns (WeSphere) to facilitate Knowledge Heritage objectives.

In this way, the WHOMER positions Digital Twins not only as mirrors of physical reality but as living repositories of meaning, enabling knowledge workers to externalize, personalize, and evolve their cognitive and cultural worlds.

## 8. Shaping Enabling Memetic Communication and Doer Spaces

This section explores how memetic boundary objects (BOs) can enable communication and co-creation across economic, architectural, and platform contexts, positioning them as key components of MDTs.

### 8.1. Complementary Contexts of Economics and Architecture

Although from distinct disciplines, Schlaile’s and Mavromatidis’s contexts complement each other. Their systemic needs provide fertile ground for applying CDTs and MDTs introduced in earlier KMS-related sections. This resonance is further strengthened as Mavromatidis’s entropic contexts also align with the findings of two PKMS-related articles [23,24]. Both authors highlight the complexities of communication and knowledge transfer across individuals, teams, stakeholders, and collectives, as depicted in Section 5’s SECI-based co-creation model. The remainder of this article focuses on the role of memetic BOs in these endeavors to further expand on Mavromatidis’s take on semiotics.

### 8.2. Atomic Units of Meaning and Boundary Objects

As briefly mentioned, mythemes, narratemes, biographemes, biobits, reputemes, memes, motifs, and topos commonly serve as fundamental ‘atomic’ elements to be repurposed as sense-giving building blocks for constructing more complex structures and guiding cultural communication. As compact carriers of meaning, they can be remixed—copied, transformed, or combined (as noted by Schlaile in Section 2)—to construct and disseminate knowledge across contexts (e.g., storytelling, sensemaking, sensegiving, knowledge systems) via methods (e.g., hierarchical structuring, sequential aggregation, relational bundling, cross-domain integration).

### 8.3. The BOTTOMS Framework

Table 4 portrays their complementing bottom-up functionalities as a special category of craft memes forming a Boundary Object Taxonomy to Omnify* Memetic Storytelling (BOTTOMS). BOTTOMS refers to a taxonomy that universalizes memetic storytelling by integrating diverse atomic units of meaning. By framing these atomic units as memetic boundary objects, BOTTOMS demonstrates how MDTs can operationalize memetic boundary objects, enabling structured communication and knowledge dissemination across diverse domains.

Eight semiotic ‘atomic’ units of meaning—structural, personal, social, cultural, and symbolic—form a unified framework for authorship and knowledge dissemination in both fiction and non-fiction. The synergies and visualizability potentials exemplified show their utility not only in the economic, architectural, and platform-related contexts discussed but also in the educational and other industrial spaces. Instantiated within a platform for knowledge co-creation, their scope differs compared to, for example, Greimas’s models referred to [31,32], Phillips’s and Huntley’s ‘Dramatica’ as a theory of and software for story [77], or GOLEM (graphs and ontologies for literary evolution models) [78].

The conceptualization of the BOTTOMS-taxonomy is one of the novel contributions of this article, but it remains to be implemented. However, its close ‘twin’, the memetic knowledge map, which further differentiates the mentioned meme clusters (content, container, context, craft, and connector), has been applied since the first PKMS prototype. Its task is to structure the authoring and storage logics and logistics, for example:

• Two Mytheme Characters [MC] (animate containers: Ulrich Schmitt and Publisher MDPI) collaborate for Biobits [BB] (connector: social/relational capital).
• MC:Schmitt starts a Mytheme Action [MA] (craft: prepares for article acceptance) for Biobits [BB] (connector: Emotional/Strategic Capital).
• A Mytheme Themes [MT] (context Biomimetics) provides MC:Schmitt an opportunity for another Biographeme [BG] (connector: research interest).
• Two key Narrateme Sources [NS] utilized are (inanimate containers: MC:Schlaile’s and MC:Mavromatidis’s publications) and form the foundation for another NS (inanimate container: MC:Schmitt’s article).
• All three NSs are linked to their respective authors for Reputemes [RP] (connector: intellectual capital and reputation/citation metrics). MC:Schmitt’s prototype as a NS:Artifact adds another Reputeme [RP] (connector: MDT system engineering expertise).
• The three NSs disseminate shared, explicit information, meanings, and substance in the form of three distinct Narrateme Payloads [NP] (content: text, tables, figures).
• Some memetic elements of the three NPs contain individual and common Motifs [MO] (sub-content: shared recurring anchors; private if still at the topos stage).
• The Topos [TO] represents the private uses and remix practices (unshared content: own creative/captured contributions to the repurposed shared MC, MA, MT, NS, NP, MO, as well as BB, BG, and RP) for memeplexes, knowledge assets, or corpora. Once these contributions are voluntarily shared, each stored topos element is categorized as its appropriate Bottoms (BO) unit.

### 8.4. Platform Integration and Cognitive Twins

The PKMS-platform-supporting BOTTOMS as a semantic web technology aims to afford a remedy to overcome the communication, monitoring, and reasoning challenges inferred in Section 4. As Schlaile explicitly refers to “compatibility relations” to represent the edges connecting network nodes [11] (pp. 48–52), it should be noted that WHOMER also allows for user-defined memetic incompatibility connectors as a further means to curate the repository’s content.

A systematic literature review already noted a “growing popularity [of] ontologically enriched DTs” among “researchers and engineers […] often called cognitive twins” which also applies to knowledge graphs (like WHOMER) “due to their expressiveness of semantic relations and their role in facilitating semantic interoperability” [79] (pp. 442, 452).

## 9. Conclusions and the Road Ahead

This section summarizes the article’s contributions, current progress, limitations, and outlines the road ahead for MDTs.

### 9.1. Purpose and Contribution

The aim of this article is to demonstrate the utility of Memetic/Metaphorical Digital Twins (MDT) as envisioned in a digital platform under development for knowledge co-creation for other disciplines and/or sectors, as exemplified for economics and architecture.

This contribution adds to a series of prior multi-disciplinary indexed publications, not unusual for “longitudinal streams of [DSR] research”. They are published at appropriate times to reflect evolving artifacts and design theories, including early visions of technology impact and studies of applied impact on users, organizations, and society. They also scrutinize existing and emerging research findings, methodologies, and practices for integration into continuous design evaluation, benchmarking, and dissemination [80] (p. 369) covering feasibility, suitability, acceptability, and theory effectiveness (a DSR paradigm which expects designs to be purposeful—both in terms of utility (a matter of content/function) and communication (a question of presentation) to an audience [81]) or, specific to this case, the applicability of its CDT and MDT components in economics and architecture, and their synergies with biomimetics.

### 9.2. Implementation and Current Progress

Inspired by Schlaile’s and Mavromatidis’s articles, a conceptualization of the BOTTOMS-taxonomy has been proposed and exemplified, which remains to be implemented. However, the overarching memetic knowledge map has been applied, and the author’s PKMS-related publications with their citing and cited references have been captured in their memetic formats in the WHOMER database together with other test-datasets, including, for example: personal contact bases and libraries; family trees; cocktail database; directories of journals, universities, cities, regions, and countries; Excellence in Research for Australia (ERA) database sets; industrial classification systems; standards, criteria, and self-assessments for a MBA program accreditation. Memetic relationships have captured all BOTTOMS elements, but they are currently only implicitly represented under the umbrella of the five c meme clusters to be explicitly re-categorized following the novel BOTTOMS taxonomy.

### 9.3. Limitations

As this platform is still under development, neither its testing in a real-world application space nor the publication of a case study is possible. While case studies, following Yin’s definition, evaluate the performance of designed objects in complex, specific, real-world settings [82], illustrative scenarios extend this scope to artifacts’ suitability/utility within a synthetic environment, which may include more generalizable or even ideal contexts [83]. This expansion bridges the gap between the natural/social and design science paradigms and accounts for DSR-limitations referred to as a “rigor-relevance dilemma” [84] (p. 1118). A review of 148 journal articles substantiated the essential necessity to rigorously evaluate DSR artifacts by employing eight evaluation methods with 2nd-ranked illustrative scenarios (14%) versus 4th-ranked case studies (5%) [83].

### 9.4. Future Developments and Road Ahead

Together, these developments demonstrate how MDTs can extend beyond economics and architecture. Further developments include AI-supported curation, personalized metrics and notifications, and expanded boundary objects to reduce the effort of creating complex documents. In this way, MDTs provide a universal framework for knowledge co-creation, extending Digital Twin typologies into cultural and conceptual domains.

## Figures and Tables

**Table 1:** Digital Twin archetypes and memetic roles.

**Archetype**	**Core Features**	**Memetic Perspective**
Industrial Digital Twins (IDT)	Establish bi-directional links between physical assets and virtual models. First-order cybernetic feedback loops (CFLs) transmit data for monitoring and prediction, while second-order CFLs involve agents managing lifecycle interventions [33].	IDTs prioritize content and container memes, encapsulating operational data streams and system knowledge for replication and reuse.
Human Digital Twins (HDT)	Represent animate agents in real space (e.g., health lifecycle management). Their virtual counterparts integrate with IDTs and other HDTs to capture personal relationships, advancing cyber-physical–social system integration (CPSS) [34].	HDTs further emphasize connector memes, embedding social relationships and linking human and machine contexts.
Cognitive Digital Twins (CDT)	Mirror individuals’ knowledge, experiences, and intellectual assets. They support private, social, educational, and professional priorities, and enable voluntary sharing for co-creation and innovation as well as cyber–physical–psycho–social integration [35].	CDTs further focus on context memes, structuring personal expertise and interpretations for collaborative knowledge generation in multi-disciplinary areas.
Memetic/Metaphorical Digital Twins (MDT)	Extend beyond technical and personal domains to represent evolving cultural knowledge and the cumulative synthesis of individuals’ cognitive provisions for knowledge heritage. MDTs emphasize the transmission, adaptation, and transformation of memes within shared knowledge ecosystems.	MDTs embody transformative memes by twinning their conceptual habitat, preserving cultural heritage while enabling dynamic co-creation across communities.

**Table 2:** Selected features according to the platform’s value propositions (summarized from a more comprehensive table [4] (p. 118)).

**Value** **Proposition**	**Memetic Role**
Absorptive Capacity (resourceful)	Memes act as living entities in the ideosphere. Skills highlighted by the Inner Development Goals (e.g., co-creation, complexity awareness, communication, connectedness) underpin the PKMS vision.
Generative Value (constructive)	Knowledge is mapped through five meme classes and numerous sub-classes, capturing both tangible and abstract entities. This structured graph supports sensemaking and relational context.
Functional Value (productive)	Fine-grained meme references enable direct linking between ideas rather than whole documents, improving retrieval, reuse, and invisible work integration.
Psychological Value (thrivable)	Meme-based boundary objects (heuristics, frameworks) guide users in navigating complex, transdisciplinary problems by re-contextualizing reusable practices.
Economic Value (viable)	PKMSs complement organizational knowledge systems by fostering connectivity and collaboration among diverse actors (ACME), encouraging participatory co-evolution.
Transformative Value (transformational)	Centralized curation preserves meme integrity, reduces redundancy, and builds a WHOMER as a long-term knowledge heritage archive.

**Table 3:** Platform’s SICECE cycle aligned to study results [63], including attribute clusters (X#) that map to tables in Sweeney’s appendix. Definitions of variables: C1—behavioral actions, C2—knowledge outcomes.

**S**eizing or Scholarly Space:	Access to external and/or PKMS’s knowledge repository (*individual acquisition from others or collective content C1b/2a*) allows—after personally assessing its fit and quality (*individual valuation C1d*)—for absorption (*individual learning C2b*), usage (*individual application C1c*), and/or further exploration (*individual performance C2d*).
**I**mbedding Space	Knowledge to be retained is captured or transferred to one’s PKMS device (*individual contribution/content C1a/2a*) and may be modified or complemented by one’s own authorship (*individual contribution/content/performance C1a/2a/2d*).
**C**ollating Space:	New associative relationships may create novel knowledge assets (*individual contribution/content/performance C1a/2a/2d*), and selected knowledge objects may be voluntarily shared (*individual contribution C1a to collective content/learning C2a/2c*).
**E**ncompassing Space:	The shared knowledge object by the PKMS community members (*platform provider’s acquisition C1b*) is to be aggregated, assessed, and curated (*provider’s valuation of communities’ collective performance C1d/2e*).
**C**urating Space	Centralized curation preserves meme integrity, reduces redundancy, and builds a WHOMER as a long-term knowledge heritage archive (*provider’s contribution/content/performance C1a/2a/2d to facilitate individual/collective content/performance C2a/2d/2e*).
**E**ffectuating Space	Added-value curation services, such as, for example, eLearning modules, boundary objects, notifications, reports, and citation/reputation metrics, are complementing the updated knowledge base accessible to the PKMS community members (*provider’s contribution/content C1a/2a).* The information processed (*provider’s performance C2d*) also includes micro-macro-micro feedback to produce self-organization and synchronization: member’s micro-behaviors, effects, or responses may result in emerging behaviors, effects, and patterns at the macro level to be fed back to the community where it may subsequently affect the member’s micro-states (*individual contribution/performance C1a/2d to collective content/performance C2a/2e to individual content/performance C2a/2d*) [64,65].

**Table 4:** Boundary Object Taxonomy to Omnify * Memetic Storytelling (bottoms).

‘Memetic BO’ Units/Domain *Source* Brief Definition of Craft Memes	Layer & Purpose *Higher-order Units* Visualizability Potentials	Synergies of Purpose (fiction/non-fiction) *Functions or Roles as Meaning Units*
Mytheme/Mythology *Levi-Strauss* [30] Atomic unit of narrative (character+action+theme)	Mythical Context for Cultural Depth and Universal Recognition *Mythic Cycles, Archetypes, Conflicts* Icons, Maps, Word Clouds	DNA or Deep Grammar of Stories *Structural logic, sequential engagement, archetypes for coherence, cohesion, comprehension, chronology, causality, conflict resolution, meaning, mission, momentum, resonance, hooks.*
Narrateme/Narrative Theory *Propp* [69,70] The smallest unit of a story for plot progression or action	Narratives for Rational Structures/Flows *Scenes, Acts, Tales, Chapters, Books, Movies* Flowcharts, Storyboards, Panels, Cartoon Strip Frames	
Biographemes & Biobits/ Biographical Media *Barthes, Moulin* [71], *Howes* [72] Minimal factual, symbolic, anecdotal elements in life story	Bs&Bs for Emotional Experience/Intimacy *Events, Episodes, Phases, Memoirs, Biographies, Chronicles, Histories* Gantt Charts, Time & Map Markers, Portraits, Sketches, Caricatures	Humanizing Facets in Characterizations *Identity for character development/perception, authenticity, authority, credibility, and personal identification.* *Life stories/recognitions also propagate culturally meaningful behavioral memes.*
Reputeme/Social Contexts *Bourdieu, Gernalzick* et al. [73] Minimal unit for reputation or social recognition	Recogs. for Social Traction/Role Models *Academic Citation/Reputation Metrics, Online Likes/Rankings* Share in Pie Charts, Color Coding	
Meme/Memetics & Culture *Dawkins* [44], *Dennett* [74] An atomic unit of cultural transmission, behavior, or idea	Cultural Memes for Viral Distribution *Memeplexes, Knowledge Assets, Corpora, Campaigns, Trends* Blueprints, Infographics, Video Clips	Memorization and Dissemination *Stickiness aids memetic replicators of culture for transmission, scalability, and virality.* *Recurring notions strengthen resonance across contexts.*
Motif Topoi/symbolic rhetorical *Aristotle* [75], *Curtius* [76] Basic recurring ideas, analogy, metaphor	Symbolic & Rhetorical Anchors (e.g., memes as living organisms) *Compelling Argumentative Reasoning* GIFs, Icons, Images, Sceneries	

* Omnify: To make universal/all-encompassing, signifying inclusivity and comprehensiveness (universalize, encompass, globalize).

## Data Availability

Data is contained within the article.

## Abbreviations

The following abbreviations are used in this manuscript:

ACME	Abilities, Contexts, Means, and Ends
BO	Boundary Object
BOTTOMS	Boundary Object Taxonomy to Omnify Memetic Storytelling
CDT	Cognitive Digital Twin
DSR	Design Science Research
DT	Digital Twin
HDT	Human Digital Twins
IDG	Inner Development Goals
IDT	Industrial Digital Twins
KM	Knowledge Management
KMS	Knowledge Management System
MDT	Memetic/Metaphorical Digital Twin
OKMS	Organizational Knowledge Management System
PKMS	Personal Knowledge Management System
SDG	Sustainable Development Goals
SECI	Nonaka’s Unified Model of Dynamic Knowledge Creation (Socialization, Externalization, Combination, Internalization)
SICECE	The Co-Creation Platform’s applied Dynamic PKMS Model (Seizing, Imbedding, Collating, Encompassing, Curating, Effectuating)
WHOMER	World Heritage of Memes Repository
